# Supplementary material for: Ribonuclease 4 Functions in Nociceptor-Mediated Nerve Homeostasis
Source: Nat Commun. 2026 Mar 24;17:2862. doi: 10.1038/s41467-026-70365-8 (PMC13022371; doi:10.1038/s41467-026-70365-8)
Supplement: Supplementary file 1 — Supplementary Information [file 41467_2026_70365_MOESM1_ESM.pdf]

Supplementary Information

Supplementary Figures:

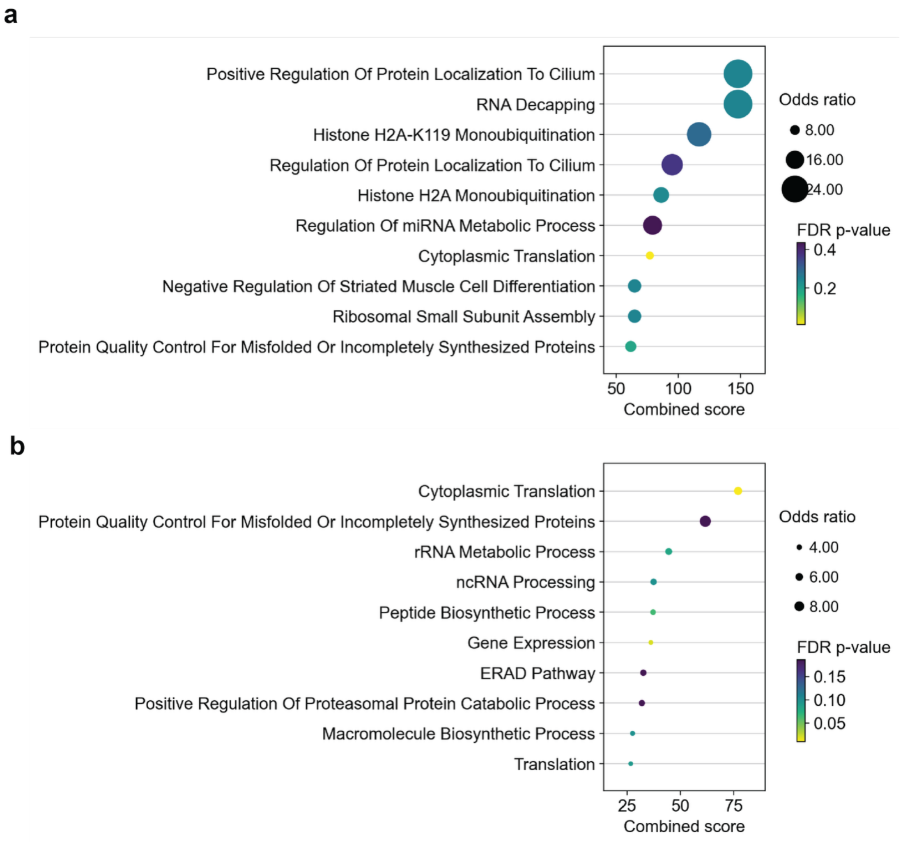

Supplementary Fig. 1: GO Biological Processes in cKO-BR

a Dot plot representing the top 10GSEA of GO biological process terms from the differentially expressed genes ordered by the combined score. **b** Dot plot representing the top 10GSEA of GO biological process terms from the differentially expressed genes ordered by the FDR p-value.

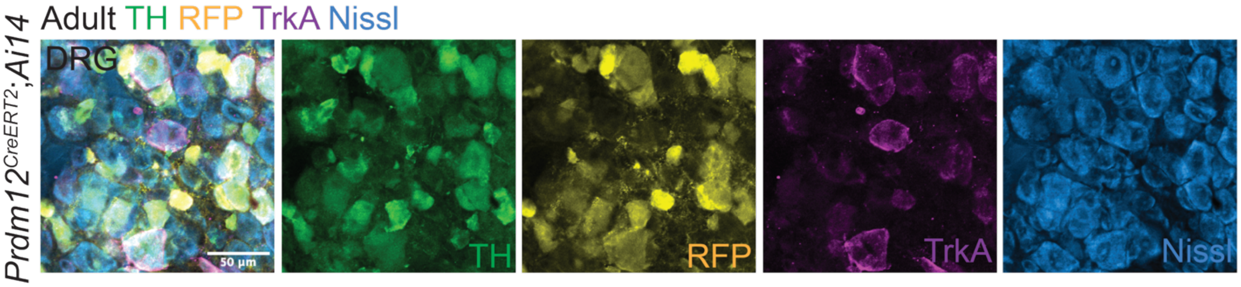

Supplementary Fig. 2: Representative Immunostaining of Adult *Prdm12*^{CreERT2};Ai14 DRG with DRG Subtype Markers

Representative images of Adult *Prdm12*^{CreERT2};Ai14 mouse DRG stained with TH, RFP, TrkA, and Nissl with a scale bar representing 50µm (total n=3 mice).

a

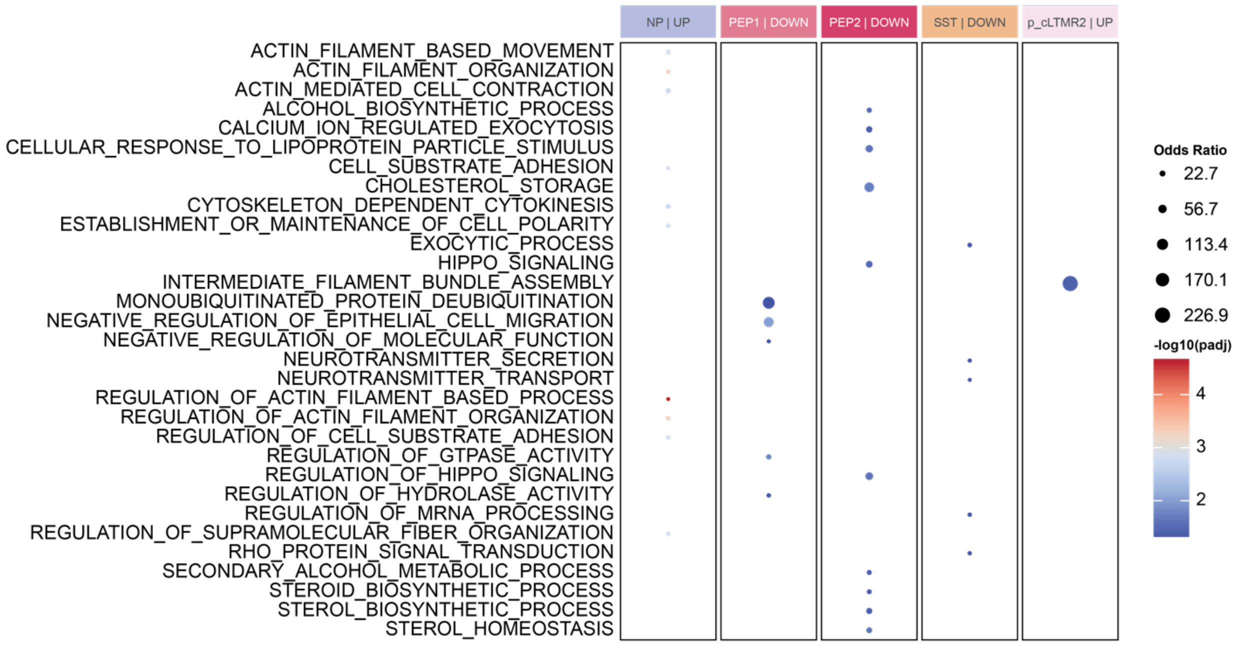

Supplementary Fig. 3: GO Biological Processes from DEGs of cKO-PR Nociceptive subtypes
a Dot plot representing the GSEA of GO biological process terms that passed the FDR=0.05 in each subtype for both significantly up and down-regulated genes.

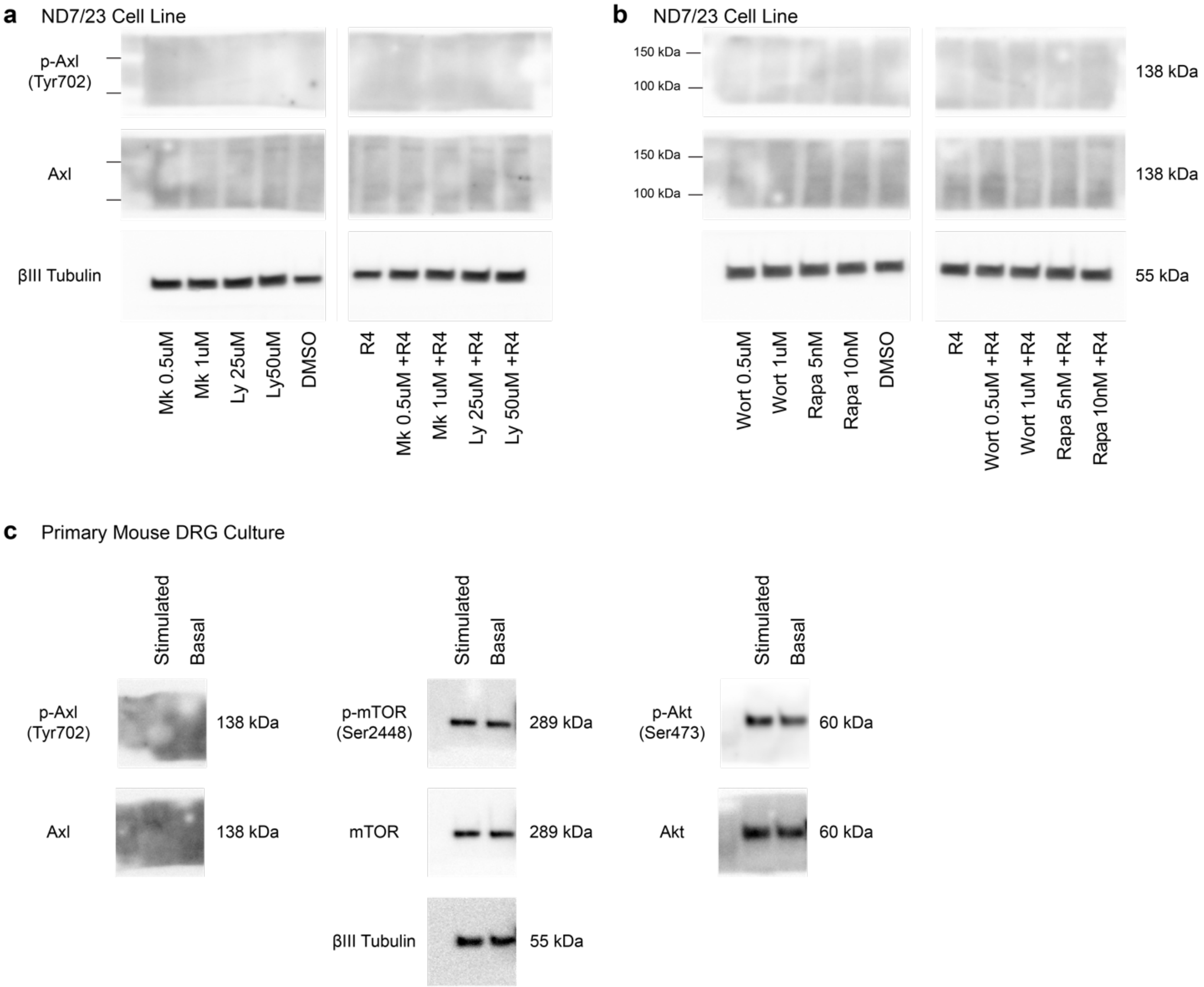

Supplementary Fig. 4: Representative Western Blots Demonstrating Absence of Axl Expression in DRG-derived Cells and the Akt-mTOR Expression in Primary Mouse Cells
a and b Representative pAxl, Axl and bIII tubulin blots from ND7/23 cell line with various inhibitor and RNase4 treatment, total n=3 independent biological replicates. Thin black lines indicate the splicing of originally non-adjacent lanes from the same blot. **c** pAxl, Axl, p-mTOR, mTOR, p-Akt, Akt and bIII tubulin blots from primary mouse DRG neuron culture under basal and capsaicin stimulated conditions. This experiment was done once for indication. Source data are provided in Supplementary Fig. 7 and 8.

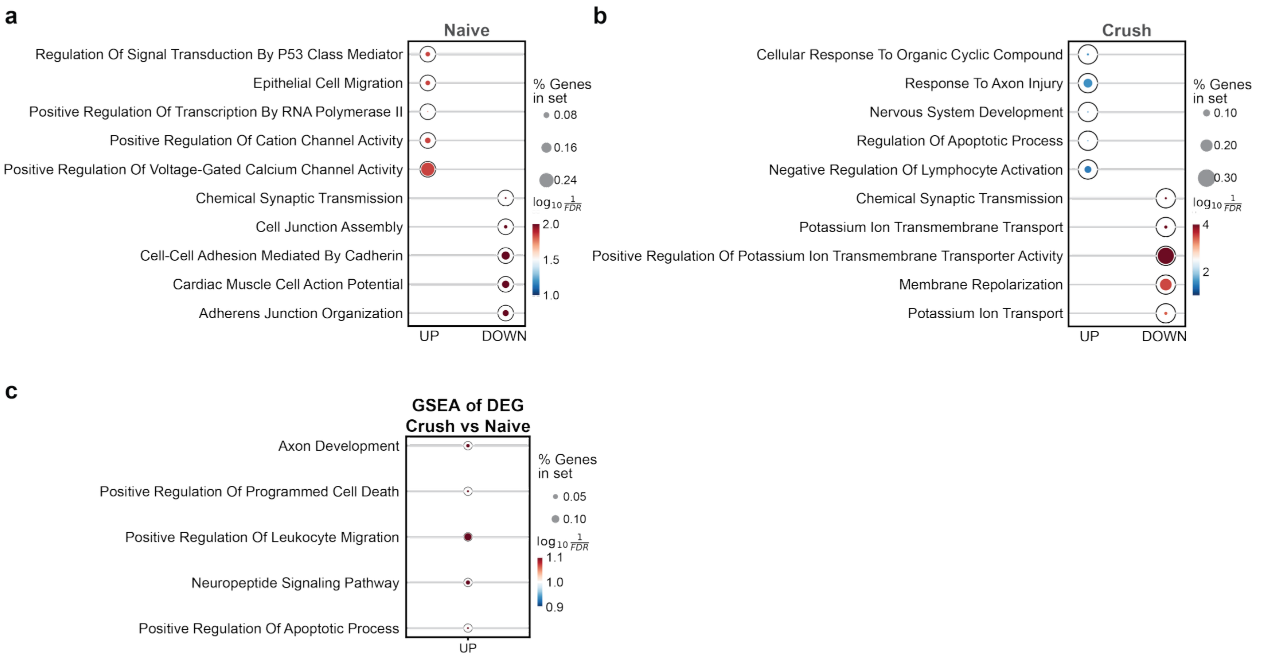

Supplementary Fig. 5: GO Biological Processes in Naive and Crush-Injured Nociceptors

a Dot plot representing the GSEA of GO biological process terms from the top 100 genes positively and negatively correlated with RNase4 in the naïve state. **b** Dot plot representing the GSEA of GO biological process terms from the top 100 genes positively and negatively correlated with RNase4 in sciatic nerve crush injury model. **c** Dot plot representing the GSEA of GO biological process terms from the top 100 upregulated genes in Crush model (Crush vs Naïve). None of the pathways from GSEA of the top 100 downregulated genes were significant.

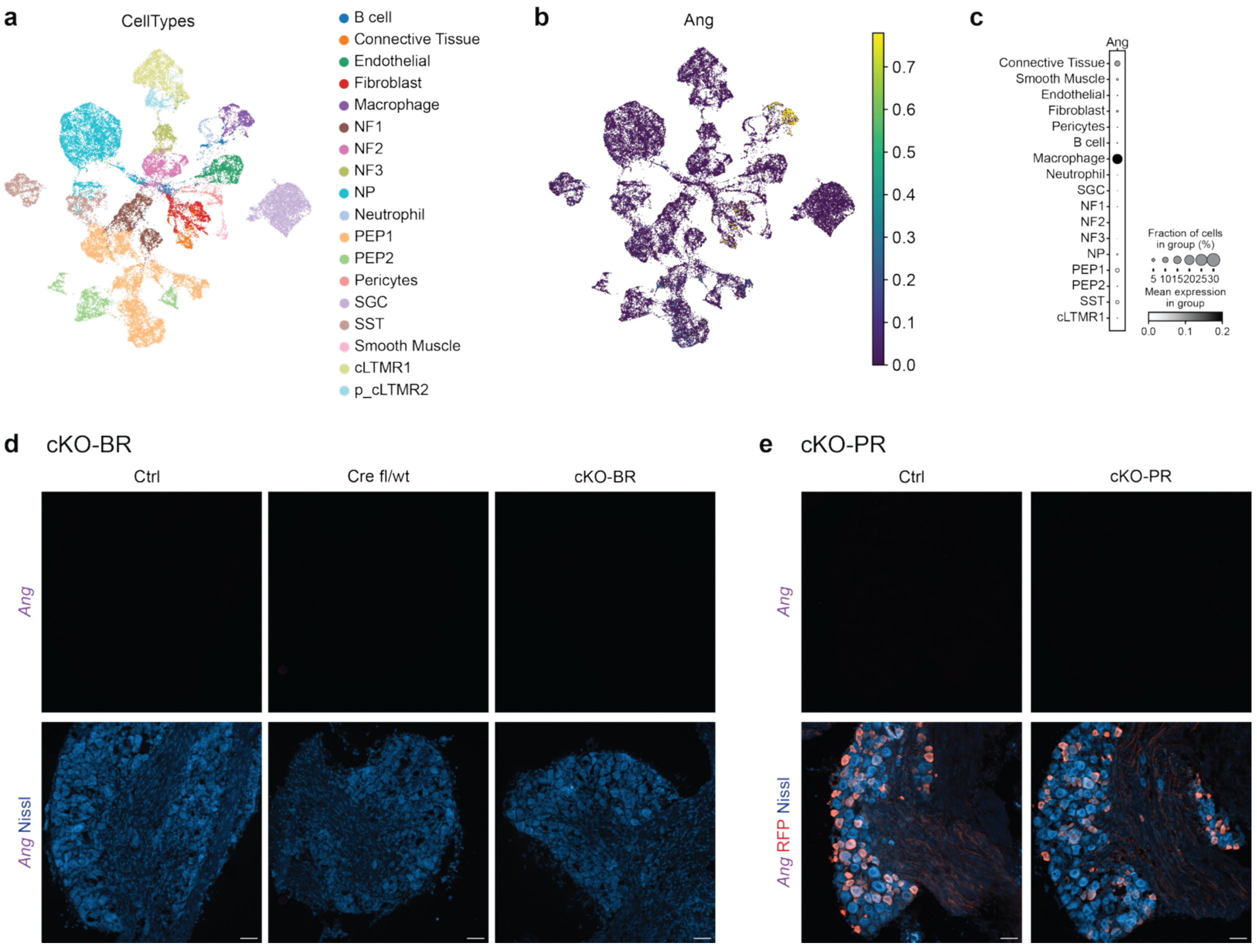

Supplementary Fig. 6: Characterization of *Angiogenin* (*RNase5*) in WT and cKO DRGs

a A UMAP plot of cell types in the DRG colored by the respective cellular identities. **b** A UMAP plot of cells in the DRG colored by the expression level of *Angiogenin* (*Ang*). **c** A dot plot of *Ang* expression level and fraction in each cellular identity within the mouse DRG. **d** Representative images from RNAscope of *Ang* in *Rnase4*$^{fl/fl}$ (Ctrl, n=3), *Baf53b*^{Cre};*Rnase4*$^{fl/wt}$ (Cre fl/wt, n=4), and *Baf53b*^{Cre};*Rnase4*$^{fl/fl}$ (cKO-BR, n=3) sections. **e** Representative images from RNAscope of *Ang* in *Prdm12*^{CreERT2};Ai14 (Ctrl, n=3) and *Prdm12*^{CreERT2};*Rnase4*$^{fl/fl}$;Ai14 (cKO-PR, n=3) sections, scale bar =50 µm.

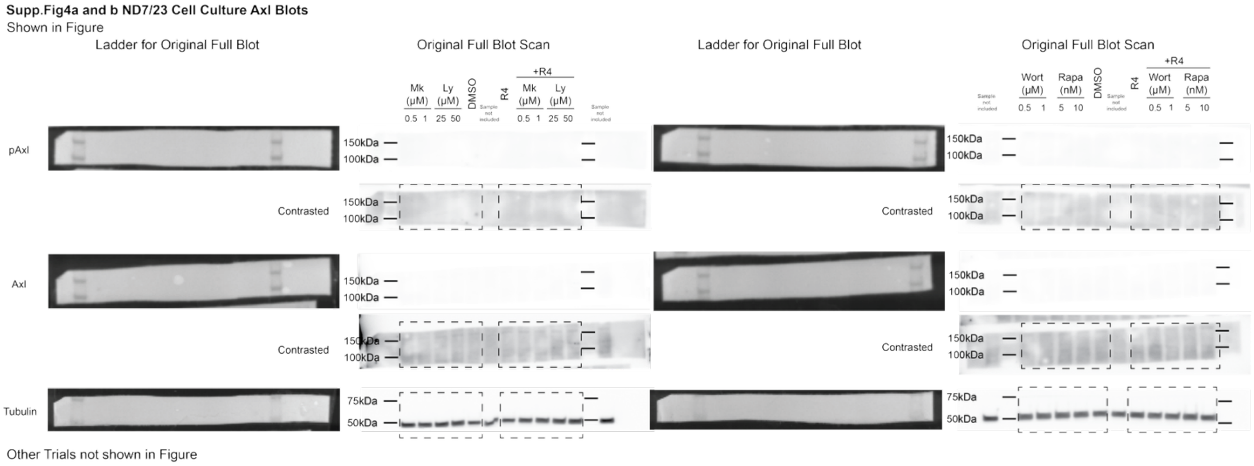

Supplementary Fig. 7: Full original blots represented in Supplementary Fig. 4a and b

Original full scan blots included in Supplementary Fig. 4a and b, black rectangles depict areas included for the Figure.

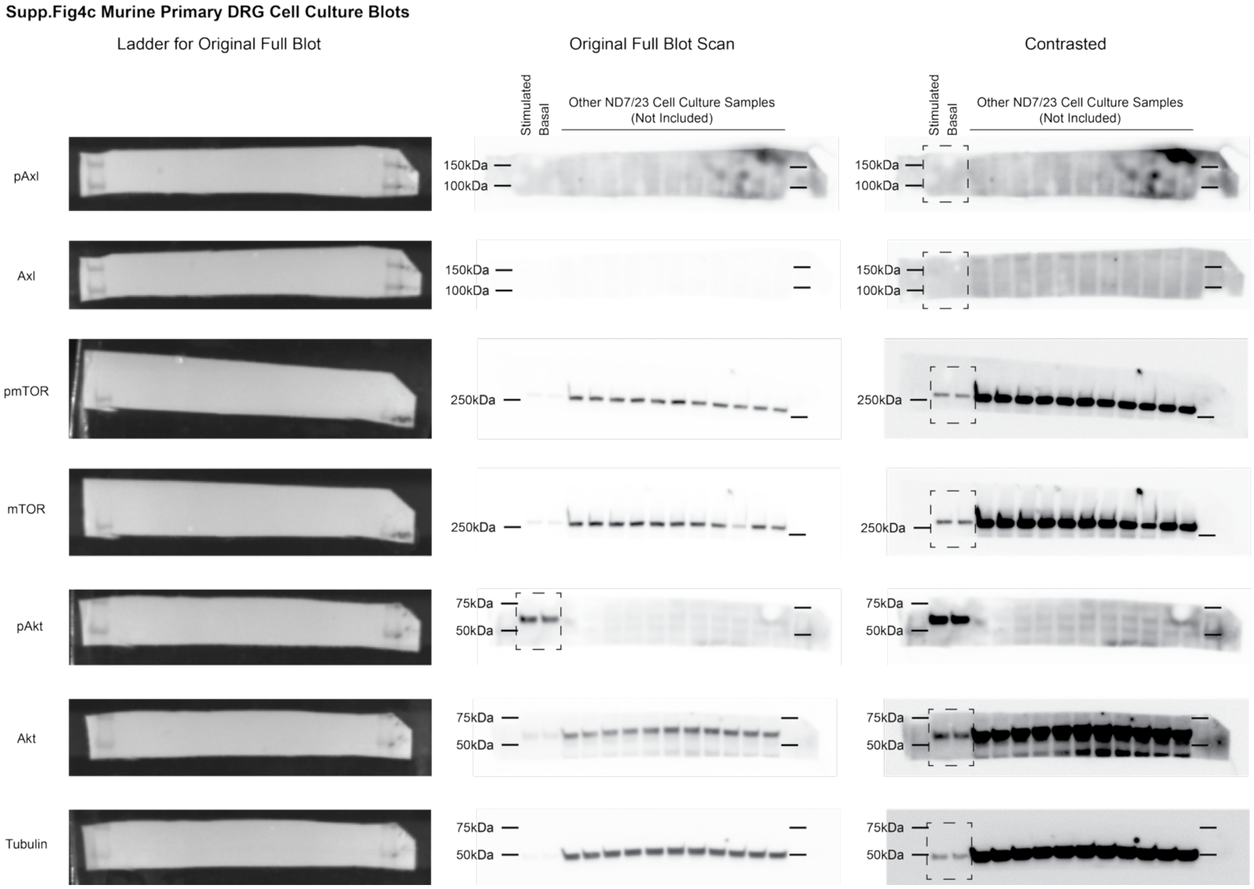

Supplementary Fig. 8: Full original blots represented in Supplementary Fig. 4c

Original full scan blots included in Supplementary Fig. 4c, black rectangles depict areas included for the Figure.

Supplementary Tables

Supplementary Table1: List of primers for genotyping with PCR gel electrophoresis.

**Line**	**Primer**
Prdm12^{CreERT2} 5'F	AGC ATG AAG TGC AAG AAC GTG
Prdm12^{CreERT2} 3'R	CAA GAG CAC TCG GAC ATT TC
Ai14 mut 5'F	CTG TTC CTG TAC GGC ATG G
Ai14 mut 3'R	GGC ATT AAA GCA GCG TAT CC
Ai14 WT 5'F	AAG GGA GCT GCA GTG GAG TA
Ai14 WT 3'R	CCG AAA ATC TGT GGG AAG TC
RNAse4$^{fl/fl}$ 5'F	CTC TCC AGA GCC CAG TCC TTA C
RNAse4$^{fl/fl}$ 3'R	GTG CAT TGC CAC CTG TCA CCT G
Baf53b^{Cre} 5'F	ACC AGG TTC GTT CAC TCA TGG
Baf53b^{Cre} 3'R	AGG CTA AGT GCC TTC TCT ACA
