# Supplementary material for: Ribonuclease 4 Functions in Nociceptor-Mediated Nerve Homeostasis
Source: Nat Commun. 2026 Mar 24;17:2862. doi: 10.1038/s41467-026-70365-8 (PMC13022371; doi:10.1038/s41467-026-70365-8)
Supplement: Supplementary file 2 — Description of Additional Supplementary Files [file 41467_2026_70365_MOESM2_ESM.pdf]

Supplementary Data 1: List of marker genes for NFs and Nociceptors identified with PyDESeq2. P-values were calculated using two-tailed Welch's t-test and the multiple hypothesis testing was corrected with Benjamini-Hochberg.

Supplementary Data 2: Differentially expressed genes from PyDESeq2 of Nociceptors from cKO-BR and cKO-PR mouse lines when compared to WT mice. P-values were calculated using two-tailed Welch's t-test and multiple hypothesis testing was corrected with Benjamini-Hochberg.

Supplementary Data 3: *Rnase4* gene co-expression network identified with Spearman's rank correlation in the nociceptors of naïve mice. Multiple hypothesis testing was corrected with Benjamini-Hochberg.

Supplementary Data 4: *Rnase4* gene co-expression network identified with Spearman's rank correlation in the nociceptors of sciatic nerve crush mice. Multiple hypothesis testing was corrected with Benjamini-Hochberg.

Supplementary Data 5: Differentially expressed genes from PyDESeq2 of Nociceptors from naïve mice compared to sciatic nerve crush mice. P-values were calculated using two-tailed Welch's t-test and multiple hypothesis testing was corrected with Benjamini-Hochberg.

Supplementary Data 6: Details and sequences used for the generation of $RNase4^{fl/fl}$ mouse line

Supplementary Data 7: Details and sequences used for the generation of $Prdm12^{CreERT2}$ mouse line
