# Supplementary material for: Ribonuclease 4 Functions in Nociceptor-Mediated Nerve Homeostasis
Source: Nat Commun. 2026 Mar 24;17:2862. doi: 10.1038/s41467-026-70365-8 (PMC13022371; doi:10.1038/s41467-026-70365-8)
Supplement: Supplementary file 8 — Supplementary Data 6 [file 41467_2026_70365_MOESM8_ESM.docx]

**Supplementary Data 6**

Sequence of the SCON (Short Conditional intrON) cassette for creating Rnase4^cko^ mouse allele

Color codes:

mRnase4 homology arms

SCON Artificial Intron cassette

Genotyping primer binding regions

Flanking sequences (uncolored)

CTGTTAGCTGTACGCCTGTCAATTAGTAAAGAGAACAGTGTTTGCTTTCAAGGAAGGCCATGTTGACAAACTAGCCACTCTCAGAGAAAGGGGTACAAAAGGGAAGAGCAGGAAGAAGGGGGAGATCTTAAGCAGAAGGAGGGGCAAGAGAGGGTTCTAAGAGTTTAGTTTGCTTTTCTCATTACCTCTCCAGAGCCCAGTCCTTACCATCCTTTCTCCTTGTTTTCTTCTCAGGCACTTTCTAGGTAATGATGGATCTACAGAGGACTCAGTCCTTGCTTCTGCTCTTGGTGCTGACCCTGCTGGGGTTAGGGCTTGTACAGCCCTCCTATGGCCAGGATCGAATGTACCAACGGTTCCTTCGACAGCATGTGGACCCTCAGGTAAGTAATAACTTCGTATAAGGTATCCTATACGAAGTTATTCTCTCTGCCTATTGGGGTTACAAGACAGGTTTAAGGAGACCAATAGAAACTGGGCATGTGGAGACAGAGAAGACTCTTGGGTTTCTGATAGGCACTGACATAACTTCGTATAAGGTATCCTATACGAAGTTATTTTCCCTCCCTCAGGTGACAGGTGGCAATGACAACTACTGCAACGTGATGATGCAGAGACGGAAGATGACTTCTGTCCAGTGCAAACGCTTCAACACCTTCATCCACGAAGACATCTGGAACATTCGTGGCATCTGCAGTACCACCAATATCCTGTGCAAGAACGGCCAGATGAACTGTCACGAAGGTGTAGTGAAGGTCACGGACTGCAGAGAGACAGGGAACTCCAAGGCCCCCAACTGTAGATACAGGGCAAGAACCAGCACTAGGCGAGTTGTCATTGCCTGTGAGGGTGACCCAGAGGTCCCAGTGCACTTTGACA
