# Supplementary material for: Ribonuclease 4 Functions in Nociceptor-Mediated Nerve Homeostasis
Source: Nat Commun. 2026 Mar 24;17:2862. doi: 10.1038/s41467-026-70365-8 (PMC13022371; doi:10.1038/s41467-026-70365-8)
Supplement: Supplementary file 9 — Supplementary Data 7 [file 41467_2026_70365_MOESM9_ESM.docx]

**Supplementary Data 7**

Prdm12^CreERT2^ mouse design details.

This is a KO/KI mouse model: it can be used as a Prdm12 knockout mouse when the allele is bred to homozygous or as a Cre driver when it is used as a heterozygous.

The mouse PRDM12 transcript (ensembl.org: ENSMUST00000113470.3) has five exons. We deleted Exons1 and 2 and inserted creERT2-sv40polyA in place of it.

The guide RNAs used were as follows:

LEFT GUIDE – GAGCCCTCCGCCGCCCATGA (cuts near the ATG in exon 1)

RIGHT GUIDE – ACCTCATGTGGGAGGTAGGC (cuts at the end of Exon 2)

Prdm12-CreERT2 SV40 pA ssDNA sequence

Homology Arms

CreERT2

SV40PolyA

Stop Codons

GACCCCTCTTCGTTGCCCACCTCTCCGGAGTCCACCTAGCCGTCCTTCGGCGCCCGCGGCGAGCCCTCCGCCGCCCatggccaatttactgaccgtacaccaaaatttgcctgcattaccggtcgatgcaacgagtgatgaggttcgcaagaacctgatggacatgttcagggatcgccaggcgttttctgagcatacctggaaaatgcttctgtccgtttgccggtcgtgggcggcatggtgcaagttgaataaccggaaatggtttcccgcagaacctgaagatgttcgcgattatcttctatatcttcaggcgcgcggtctggcagtaaaaactatccagcaacatttgggccagctaaacatgcttcatcgtcggtccgggctgccacgaccaagtgacagcaatgctgtttcactggttatgcggcggatccgaaaagaaaacgttgatgccggtgaacgtgcaaaacaggctctagcgttcgaacgcactgatttcgaccaggttcgttcactcatggaaaatagcgatcgctgccaggatatacgtaatctggcatttctggggattgcttataacaccctgttacgtatagccgaaattgccaggatcagggttaaagatatctcacgtactgacggtgggagaatgttaatccatattggcagaacgaaaacgctggttagcaccgcaggtgtagagaaggcacttagcctgggggtaactaaactggtcgagcgatggatttccgtctctggtgtagctgatgatccgaataactacctgttttgccgggtcagaaaaaatggtgttgccgcgccatctgccaccagccagctatcaactcgcgccctggaagggatttttgaagcaactcatcgattgatttacggcgctaaggatgactctggtcagagatacctggcctggtctggacacagtgcccgtgtcggagccgcgcgagatatggcccgcgctggagtttcaataccggagatcatgcaagctggtggctggaccaatgtaaatattgtcatgaactatatccgtaacctggatagtgaaacaggggcaatggtgcgcctgctggaagatggcgatctcgagccatctgctggagacatgagagctgccaacctttggccaagcccgctcatgatcaaacgctctaagaagaacagcctggccttgtccctgacggccgaccagatggtcagtgccttgttggatgctgagccccccatactctattccgagtatgatcctaccagacccttcagtgaagcttcgatgatgggcttactgaccaacctggcagacagggagctggttcacatgatcaactgggcgaagagggtgccaggctttgtggatttgaccctccatgatcaggtccaccttctagaatgtgcctggctagagatcctgatgattggtctcgtctggcgctccatggagcacccagtgaagctactgtttgctcctaacttgctcttggacaggaaccagggaaaatgtgtagagggcatggtggagatcttcgacatgctgctggctacatcatctcggttccgcatgatgaatctgcagggagaggagtttgtgtgcctcaaatctattattttgcttaattctggagtgtacacatttctgtccagcaccctgaagtctctggaagagaaggaccatatccaccgagtcctggacaagatcacagacactttgatccacctgatggccaaggcaggcctgaccctgcagcagcagcaccagcggctggcccagctcctcctcatcctctcccacatcaggcacatgagtaacaaaggcatggagcatctgtacagcatgaagtgcaagaacgtggtgcccctctatgacctgctgctggaggcggcggacgcccaccgcctacatgcgcccactagccgtggaggggcatccgtggaggagacggaccaaagccacttggccactgcgggctctacttcatcgcattccttgcaaaagtattacatcacgggggaggcagagggtttccctgccacagctTGATAACTAAGTAAGGATCCAGACATGATAAGATACATTGATGAGTTTGGACAAACCACAACTAGAATGCAGTGAAAAAAATGCTTTATTTGTGAAATTTGTGATGCTATTGCTTTATTTGTAACCATTATAAGCTGCAATAAACAAGTTggcgggagctttggaaagggattccagttggcaggtgggttgtatccactggcggcagaaaggaactctgtctggccc
