# Supplementary material for: Ribonuclease 4 Functions in Nociceptor-Mediated Nerve Homeostasis
Source: Nat Commun. 2026 Mar 24;17:2862. doi: 10.1038/s41467-026-70365-8 (PMC13022371; doi:10.1038/s41467-026-70365-8)
Supplement: Supplementary file 10 — Reporting Summary [file 41467_2026_70365_MOESM10_ESM.pdf]

nature portfolio

Corresponding author(s): Saida Hadjab

Last updated by author(s): Jan 30, 2026

Reporting Summary

Nature Portfolio wishes to improve the reproducibility of the work that we publish. This form provides structure for consistency and transparency in reporting. For further information on Nature Portfolio policies, see our Editorial Policies and the Editorial Policy Checklist.

Statistics

For all statistical analyses, confirm that the following items are present in the figure legend, table legend, main text, or Methods section.

n/a	Confirmed	
☐	☒	The exact sample size (*n*) for each experimental group/condition, given as a discrete number and unit of measurement
☐	☒	A statement on whether measurements were taken from distinct samples or whether the same sample was measured repeatedly
☐	☒	The statistical test(s) used AND whether they are one- or two-sided<br>*Only common tests should be described solely by name; describe more complex techniques in the Methods section.*
☐	☒	A description of all covariates tested
☐	☒	A description of any assumptions or corrections, such as tests of normality and adjustment for multiple comparisons
☐	☒	A full description of the statistical parameters including central tendency (e.g. means) or other basic estimates (e.g. regression coefficient) AND variation (e.g. standard deviation) or associated estimates of uncertainty (e.g. confidence intervals)
☐	☒	For null hypothesis testing, the test statistic (e.g. *F*, *t*, *r*) with confidence intervals, effect sizes, degrees of freedom and *P* value noted<br>*Give P values as exact values whenever suitable.*
☒	☐	For Bayesian analysis, information on the choice of priors and Markov chain Monte Carlo settings
☒	☐	For hierarchical and complex designs, identification of the appropriate level for tests and full reporting of outcomes
☒	☐	Estimates of effect sizes (e.g. Cohen's *d*, Pearson's *r*), indicating how they were calculated

Our web collection on statistics for biologists contains articles on many of the points above.

Software and code

Policy information about availability of computer code

Data collection	Smart-seq3 express: zUMIs v2.9.7 software was used to process the raw sequencing files to generate the count matrices.<br>10x Genomics: Cell Ranger v9.0.1 software was used to process the raw sequencing files to generate the count matrices.<br>Immunofluorescent images were taken using Zeiss LSM 800 or Zeiss LSM 800 Airyscan confocal microscopes.<br>A light sheet microscope (Ultramicroscope II, Lavision Biotec, Bielefeld, Germany) and the ImspectorTM 347 software were used for whole embryo volume imaging.<br>For CryoEM: a HT7700 transmission electron microscope (Hitachi High-Technologies) was used at 80 kV and digital images were acquired using a 2kx2k Veleta CCD camera (Olympus Soft Imaging Solutions).
Data analysis	Immunofluorescent images were analyzed using ImageJ version 1.54f.<br>The serials of 16-bit uncompressed tif images from whole embryo volume imaging were converted to IMS file using the Imaris File Converter 9.7.2 program (Bitplane, UK), and the 3D vision of acquisitions was reconstructed in the Imaris 9.7.2.<br>CryoEM images were analyzed using the Napari software platform.<br>Single cell data were analyzed using Python 3 version 3.11.9 with the following packages:<br>scanpy (1.10.2)<br>scipy (1.14)<br>numpy (1.26.4)<br>scikit-learn (1.5.1)

For manuscripts utilizing custom algorithms or software that are central to the research but not yet described in published literature, software must be made available to editors and reviewers. We strongly encourage code deposition in a community repository (e.g. GitHub). See the Nature Portfolio guidelines for submitting code & software for further information.

Data

Policy information about availability of data

All manuscripts must include a data availability statement. This statement should provide the following information, where applicable:

- Accession codes, unique identifiers, or web links for publicly available datasets
- A description of any restrictions on data availability
- For clinical datasets or third party data, please ensure that the statement adheres to our policy

The sequencing data and count matrices generated from this study have been deposited in the Gene Expression Omnibus (GEO) database under accession code GSE253345.

Research involving human participants, their data, or biological material

Policy information about studies with human participants or human data. See also policy information about sex, gender (identity/presentation), and sexual orientation and race, ethnicity and racism.

Reporting on sex and gender	*Use the terms sex (biological attribute) and gender (shaped by social and cultural circumstances) carefully in order to avoid confusing both terms. Indicate if findings apply to only one sex or gender; describe whether sex and gender were considered in study design; whether sex and/or gender was determined based on self-reporting or assigned and methods used.* *Provide in the source data disaggregated sex and gender data, where this information has been collected, and if consent has been obtained for sharing of individual-level data; provide overall numbers in this Reporting Summary. Please state if this information has not been collected.* *Report sex- and gender-based analyses where performed, justify reasons for lack of sex- and gender-based analysis.*
Reporting on race, ethnicity, or other socially relevant groupings	*Please specify the socially constructed or socially relevant categorization variable(s) used in your manuscript and explain why they were used. Please note that such variables should not be used as proxies for other socially constructed/relevant variables (for example, race or ethnicity should not be used as a proxy for socioeconomic status).* *Provide clear definitions of the relevant terms used, how they were provided (by the participants/respondents, the researchers, or third parties), and the method(s) used to classify people into the different categories (e.g. self-report, census or administrative data, social media data, etc.)* *Please provide details about how you controlled for confounding variables in your analyses.*
Population characteristics	*Describe the covariate-relevant population characteristics of the human research participants (e.g. age, genotypic information, past and current diagnosis and treatment categories). If you filled out the behavioural & social sciences study design questions and have nothing to add here, write "See above."*
Recruitment	*Describe how participants were recruited. Outline any potential self-selection bias or other biases that may be present and how these are likely to impact results.*
Ethics oversight	*Identify the organization(s) that approved the study protocol.*

Note that full information on the approval of the study protocol must also be provided in the manuscript.

Field-specific reporting

Please select the one below that is the best fit for your research. If you are not sure, read the appropriate sections before making your selection.

☒ Life sciences ☐ Behavioural & social sciences ☐ Ecological, evolutionary & environmental sciences

For a reference copy of the document with all sections, see nature.com/documents/nr-reporting-summary-flat.pdf

Life sciences study design

All studies must disclose on these points even when the disclosure is negative.

Sample size	For behaviour tests, 6 animals including 3 male 3 females per group are employed to observe significant differences among different groups.<br>For EM imaging, 3-4 mice per group were taken for analysis.<br>For RNAscope or immunostaining, a minimal of 3 mice per group (minimal of 3 DRGs per mice) were taken for analysis.<br>For iDISCO volume imaging 1 embryo was used.<br>Smart-seq3xpress single-nucleus RNA-seq: 4 mice were included to generate data.<br>10x Genomics single-nucleus RNA-seq: 6 mice (3 per genotype) were included to generate data.<br>No formal statistical methods were used to predetermine sample size. Sample sizes were chosen based on prior experience and established practice in the field and were sufficient to support statistical analyses and reproducible results.
Data exclusions	One control mouse was excluded from EM analysis due to overt abnormalities in nerve structure.
Replication	Smart-seq3xpress single-nucleus RNA-seq: The nuclei were pooled from 4 mice to generate 2 plates of smart seq3 express data.<br>10x Genomics single-nucleus RNA-seq: The nuclei were pooled from 3 mice to generate to 1 pooled run for each condition (6 mice in total for 2 conditions).

For behaviour tests, 6 animals including 3 male 3 females per group are employed to observe significant differences among different groups.
For electron microscopy (EM) analyses, 3 cKO-PR mice and 4 control mice were used, with 3–18 images acquired per mouse for each analysis.
For iDISCO volume imaging, 1 embryo was used, where results could be further confirmed on sections with RNAscope or immunostaining.
For RNAscope or immunostaining, a minimal of 3 mice per group (minimal of 3 DRGs per mice) were taken for analysis.
All experiments were replicated successfully, with no major discrepancies between independent replications.

Randomization

Mice were allocated to different experimental groups based on their randomly assigned ID by the experimenter with considerations for matching sex and age.

Blinding

No blinding was done for single-nucleus data.
For animal experiments, investigators were blinded to group allocation during animal care, housing (cage maintenance), and behavioral testing. Group allocation was not blinded during genotype- and sex-based assignment, as equal numbers of male and female mice from each genotype were required for the experimental design. Surgical procedures and tissue collection were alternated across genotypes and sexes to minimize potential time-dependent biases.

Reporting for specific materials, systems and methods

We require information from authors about some types of materials, experimental systems and methods used in many studies. Here, indicate whether each material, system or method listed is relevant to your study. If you are not sure if a list item applies to your research, read the appropriate section before selecting a response.

Materials & experimental systems

n/a	Involved in the study	
☐	☒	Antibodies
☐	☒	Eukaryotic cell lines
☒	☐	Palaeontology and archaeology
☐	☒	Animals and other organisms
☒	☐	Clinical data
☒	☐	Dual use research of concern
☒	☐	Plants

Methods

n/a	Involved in the study	
☒	☐	ChIP-seq
☐	☒	Flow cytometry
☒	☐	MRI-based neuroimaging

Antibodies

Antibodies used

For FACS: anti-NeuN PE conjugated antibody (1:500 FCMAB317PE, Merck)
For IHC: 1:400 TH (Pel-Freez, P40101), 1:400 TrkA (R&D Systems, AF1056), 1:250 NF200 (Millipore, AB1991), 1:250 peripherin (Abcam, ab39374), 1:200 CGRP (Immunostar, AB_572217), 1:500 rabbit anti-PV (Swant, PV27a) and 1:500 rabbit anti-RFP (Rockland, 600-401-379). Isolectin B4 conjugated with fluorescein was added at 1:100 together with primary antibodies were used. donkey anti-rabbit IgG AF 488 (1:1000, A-21206, Invitrogen), donkey anti-rabbit IgG 555 (1:1000, A-31572, Invitrogen) and donkey anti-goat IgG AF 647 (A21447, Invitrogen) secondary antibodies were used.
For Western Blot the following antibodies were used at a concentration of 1:1000: rabbit anti-Axl (CST, 8661), rabbit anti-phospho-Axl Tyr702 (CST, 5724), rabbit anti-mTOR (CST, 2983), rabbit anti-phospho-mTOR Ser2448 (CST, 5536), rabbit anti-Akt (CST, 9271), rabbit anti-phospho-Akt (CST, 9272) and mouse anti-βIII tubulin (Promega, G712A). HRP-linked anti-rabbit IgG (CST, 7074) or anti-mouse IgG (CST, 7076) were applied at 1:3000.

Validation

The primary antibodies used in this study were validated either by the manufacturer or through previous publications. The manufacturer's data on validation can be found on their website or product datasheet, which provides details on specificity and cross-reactivity. In addition, for antibodies used in previous studies, validation was provided by our lab and has been published (Techameena et al., Nat Commun. 2024 Oct 4;15(1):8585. doi: 10.1038/s41467-024-52052-8.)

Eukaryotic cell lines

Policy information about cell lines and Sex and Gender in Research

Cell line source(s)

The cell line was commercially available and ordered from SIGMA-ALDRICH SWEDEN AB with Catalogue No. 92090903.

Authentication

SIGMA-ALDRICH SWEDEN AB

Mycoplasma contamination

Confirm that all cell lines tested negative for mycoplasma contamination OR describe the results of the testing for mycoplasma contamination OR declare that the cell lines were not tested for mycoplasma contamination.

Commonly misidentified lines
(See ICLAC register)

Name any commonly misidentified cell lines used in the study and provide a rationale for their use.

Animals and other research organisms

Policy information about studies involving animals; ARRIVE guidelines recommended for reporting animal research, and Sex and Gender in Research

Laboratory animals	Adult male and female C57BL/6 mice (7–16 weeks old) and embryos (embryonic days 15.5–17.5) were used in this study. The strains included wild-type, Rnase4fl/fl, Bafcre;Rnase4fl/fl, Prdm12CreERT2;Ai14, and Prdm12CreERT2;Rnase4fl/fl of the C57BL/6J background.
Wild animals	No wild animals were used in this study.
Reporting on sex	Animals of either sex were used in this study.
Field-collected samples	No field-collected animals were used in this study.
Ethics oversight	All animal work was performed in accordance with the national guidelines and approved by the local ethics committee of Stockholm, Stockholms Norra djurförsöksetiska nämnd.

Note that full information on the approval of the study protocol must also be provided in the manuscript.

Plants

Seed stocks	*Report on the source of all seed stocks or other plant material used. If applicable, state the seed stock centre and catalogue number. If plant specimens were collected from the field, describe the collection location, date and sampling procedures.*
Novel plant genotypes	*Describe the methods by which all novel plant genotypes were produced. This includes those generated by transgenic approaches, gene editing, chemical/radiation-based mutagenesis and hybridization. For transgenic lines, describe the transformation method, the number of independent lines analyzed and the generation upon which experiments were performed. For gene-edited lines, describe the editor used, the endogenous sequence targeted for editing, the targeting guide RNA sequence (if applicable) and how the editor was applied.*
Authentication	*Describe any authentication procedures for each seed stock used or novel genotype generated. Describe any experiments used to assess the effect of a mutation and, where applicable, how potential secondary effects (e.g. second site T-DNA insertions, mosiacism, off-target gene editing) were examined.*

Flow Cytometry

Plots

Confirm that:

- [x] The axis labels state the marker and fluorochrome used (e.g. CD4-FITC).
- [x] The axis scales are clearly visible. Include numbers along axes only for bottom left plot of group (a 'group' is an analysis of identical markers).
- [x] All plots are contour plots with outliers or pseudocolor plots.
- [x] A numerical value for number of cells or percentage (with statistics) is provided.

Methodology

Sample preparation	DRG nuclei were dissociated as mentioned in Methods section in Fluorescent-activated nuclei sorting paragraph.
Instrument	BD instruments. BD FACSDiva 8.0.2 and BD Influx were used.
Software	FlowJo v10 (BD) and BD FACS Sortware 1.2.0/142/Utopex 1.2.0.108
Cell population abundance	The cell population abundance of the DAPI+ singlet cells were 52.8% and 51% from the total population
Gating strategy	DAPI-positive single nuclei were selected based on observed intensity and ratio of SSC-H/SSC-A. NeuN-positive nuclei were further selected to gate neuronal cells.

- [x] Tick this box to confirm that a figure exemplifying the gating strategy is provided in the Supplementary Information.
